# Use of argatroban in combination with nafamostat mesilate in open-heart surgery for a pediatric patient with heparin-induced thrombocytopenia type II: a case report

**DOI:** 10.1186/s40981-020-0310-6

**Published:** 2020-01-13

**Authors:** Shuji Kawamoto, Eriko Kusudo, Kazuhiko Fukuda

**Affiliations:** Department of Anesthesia, Kyoto University Hospital, 54, Shogoinkawahara-cho, Sakyo-ku, Kyoto, 606-8507 Japan

**Keywords:** Argatroban, Nafamostat mesilate, Heparin-induced thrombocytopenia type II (HIT II), Celite, Kaolin, Activated coagulation time (ACT)

## Abstract

**Background:**

Heparin-induced thrombocytopenia type II (HIT II) is a rare, immune-mediated complication of heparin therapy and can cause life-threatening thromboembolism. However, perioperative anticoagulation therapy for patients with a complication of HIT II has not been established.

**Case presentation:**

A 6-year-old boy with tetralogy of Fallot underwent radical intracardiac repair with administration of argatroban at 1 year old due to positive HIT antibody. Reoperation was scheduled for pulmonary valve insufficiency, using argatroban and nafamostat mesilate as anticoagulants. Argatroban has a long onset time and the activated coagulation time (ACT) requires 7–26 h to return to the preadministration level, making hemorrhage control difficult, while half-life of nafamostat mesilate is shorter than that of argatroban. Celite ACT reflects the effects of both argatroban and nafamostat mesilate, but kaolin ACT reflects only the effect of argatroban. Due to the early termination of argatroban administration based on Celite and kaolin ACTs, ACT recovered to ≤ 200 s at 5 h after the end of argatroban administration.

**Conclusion:**

Celite and kaolin ACTs can be used as markers to obtain close control of the required dose of argatroban in combination with nafamostat mesilate for the management of HIT II patients.

## Background

Heparin-induced thrombocytopenia type II (HIT II) is a serious side effect caused by use of heparin [1]. Perioperative anticoagulation therapy for patients with a complication of HIT II has not been established [1], and the direct thrombin inhibitor argatroban is the only drug approved in 2008 as a treatment for HIT II in Japan. However, since there are no antagonists for argatroban, management with this drug is difficult in many cases. In contrast, control with nafamostat mesilate is easier because of its short half-life (23.1 min), although its anticoagulant effect is lower than that of argatroban [2, 3]. In this report, we discuss the utility of concomitant use of argatroban and nafamostat mesilate based on the Celite activated coagulation time (ACT) and kaolin ACT, which do and do not reflect the effect of nafamostat mesilate, respectively [4], in open-heart surgery for a pediatric patient with HIT II.

## Case presentation

The patient was a 6-year-old boy of height 107.7 cm and weight 17.7 kg. He was born by scheduled Cesarean delivery after gestation of 38 weeks and 2 days, with weight 3026 g and height 45.5 cm, because his mother was after hysteromyctomy. At 1 day old, he was diagnosed with tetralogy of Fallot (TOF), and he received palliative surgery at another hospital at 6 weeks. At 1 year, more than 50% thrombocytopenia and upper extremity movement disorder appeared after catheterization, and he was diagnosed with HIT II because his functional assay was positive for HIT antibodies. Therefore, radical intracardiac repair with administration of argatroban was performed at the hospital. After this surgery, aortic regurgitation and residual defect of the ventricular septum were confirmed. Follow-up observation was performed for these symptoms, in addition to pulmonary stenosis and regurgitation, which were associated with TOF.

**Fig. 1:**
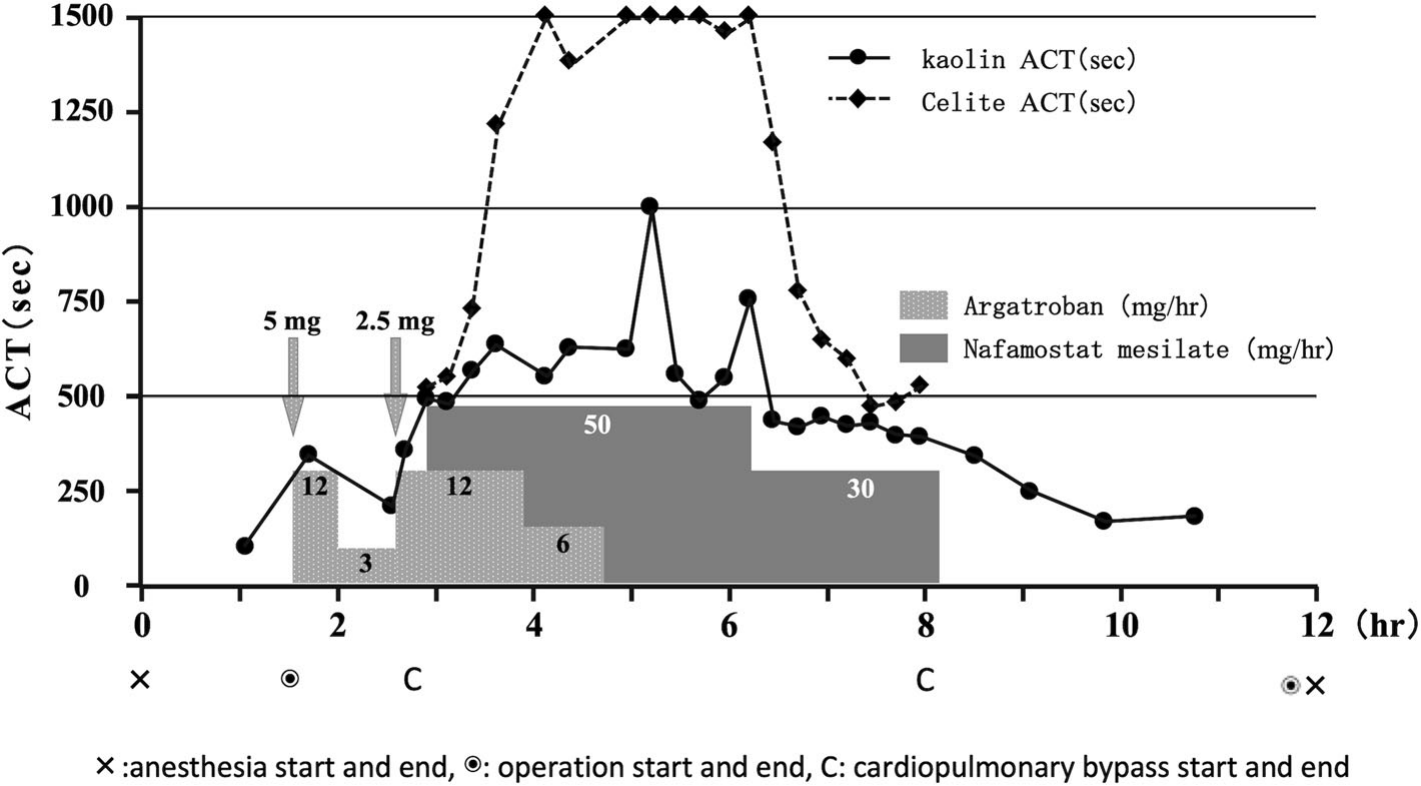
Course of the patient during surgery

Based on a desire for treatment at our hospital, the patient underwent a cardiac catheter test, which showed progression of pulmonary valve insufficiency. Thus, reoperation was scheduled for aortic valvuloplasty, pulmonary valve replacement, and repair of ventricular septal defect. Anesthesia was performed with slow induction of sevoflurane and maintained with fentanyl and sevoflurane/midazolam. No abnormalities were found on the coagulation test and HIT antibody had become negative, but argatroban and nafamostat mesilate were used as anticoagulants because some reports suggest that HIT II can redevelop due to heparin re-administration [1]. During surgery, we measured Celite ACT and kaolin ACT, which do and do not reflect the effect of nafamostat mesilate, respectively (Fig. 1). Physiological saline was used for the blood pressure line. The blood samples were collected from the arterial pressure line placed in the radial artery, but from the blood supply circuit only during cardiopulmonary bypass, because blood in the blood supply circuit most reflects the activation of the coagulation system by the cardiopulmonary bypass circuit. Therefore, there may be slight differences in ACT measurement values depending on the sampling line.

After thoracotomy, a 5-mg argatroban bolus was administered and continuous argatroban was started at 12 mg/h, which was then decreased to 3 mg/h because the activated partial thromboplastin time (APTT) was 136.6 s. However, since kaolin ACT decreased from 352 to 217 s, the dose was increased again to 12 mg/h and bolus administration of 2.5 mg was added. After kaolin ACT reached 501 s and the patient was placed on cardiopulmonary bypass, continuous administration of nafamostat mesilate was started at 50 mg/h, concomitantly with argatroban. ACT was continuously prolonged, and Celite ACT and kaolin ACT were 1500 and 600 s, respectively, at 3.5 h before weaning from cardiopulmonary bypass. Therefore, administration of argatroban was discontinued after the dose was decreased by half. ACT continued to be prolonged, although with some shortening, and thus the dose of nafamostat mesilate was decreased to 30 mg/h until weaning from cardiopulmonary bypass. Upon weaning, Celite ACT and kaolin ACT were 533 and 399 s, respectively.

ACT decreased to ≤ 200 s (177 s) for the first time at 5 h and 1.5 h after discontinuation of argatroban and nafamostat mesilate, respectively. Operation and anesthesia times were 10 h 9 min and 11 h 36 min, respectively. Intraoperative hemorrhage was about 1000 ml, and 560 ml of red blood cells (RBCs), 520 ml of fresh frozen plasma (FFP), and 250 ml of platelet concentrate (PC) were transfused. There was no significant postoperative bleeding. Extubation was performed on the day after surgery and the patient was discharged on postoperative day 11.

## Discussion

HIT II is a serious side effect of heparin that may cause thrombocytopenia and thromboembolism when platelet factor 4/heparin complex and HIT antibody form an immune complex to activate blood platelets [1]. Re-administration of heparin has been contraindicated for HIT II patients, but HIT antibody is transient and becomes negative in about 50 to 85 days [5]. Thus, an increasing number of reports suggest that reuse of heparin may not necessarily cause redevelopment of HIT II after HIT antibody becomes negative [6, 7]. However, since redevelopment of HIT II has been reported [1], we did not use heparin in our case. In the treatment of HIT II, when APTT is used as an index, it is often adjusted to 1.5 to 3.0 times the pre-dose value, but there is no clear standard for APTT and ACT in heart surgery using cardiopulmonary bypass [8]. In this case, argatroban was titrated to achieve a target APTT of 60 to 100 s, and ACT was maintained for 400 s or more during cardiopulmonary bypass as in the normal heart surgery.

Argatroban, a direct thrombin inhibitor that binds avidly and reversibly to the catalytic site of thrombin and that does not require other cofactors to exert its antithrombotic action, is often used in open-heart surgery for patients with HIT II [9, 10]. After initial bolus administration at 0.1–0.3 mg/kg and continuous dosing at 5–10 μg/kg/min during cardiopulmonary bypass, additional bolus doses and an increase in the continuous dose are often required to achieve the target ACT [11, 12]. Similarly, in our case, additional administration was required to achieve the target ACT after continuous dosing at about 11.3 μg/kg/min after bolus administration of about 0.3 mg/kg. In all previously reported cases of open-heart surgery in pediatric patients with HIT II, ACT far exceeded the target level after additional administration of argatroban based on ACT [13]. In addition, many reports suggest that 7–26 h are required for ACT to return to the preadministration level, and intra- and postoperative recovery of blood coagulation ability is prolonged, causing increased bleeding and blood transfusion volumes [9, 11, 14]. This may be because argatroban has an onset time of as long as 30 min, and a similarly long half-life of about 30 min. Unlike heparin, there are no antagonists for argatroban, and thus its effects cannot be antagonized upon excessive administration. For these reasons, we decided to use argatroban concomitantly with nafamostat mesilate, which has a shorter half-life (23 min) than that of argatroban. Nafamostat mesilate is inactivated due to hydrolysis by carboxylesterase which is present in blood and liver and the short duration of action is quite favorable for use with an extracorporeal circulation system [2, 3]. On the other hand, nafamostat mesilate is insufficient to inhibit extrinsic coagulation, and when used alone for extracorporeal circulation, a large amount of thrombus may be formed in the reservoir [15]. Therefore, we also used Celite ACT, which reflects the effect of both argatroban and nafamostat mesilate, and kaolin ACT, which does not reflect the effect of nafamostat mesilate due to its adsorption [4], as markers of the anticoagulant effects of only argatroban, and as a basis for discontinuation of continuous administration of argatroban. Using this approach, ACT recovered to ≤ 200 s comparatively early, at 5 h after discontinuation of argatroban. Concomitant argatroban and nafamostat mesilate have been used only in one case for open-heart surgery for HIT II patients [16], but in no pediatric cases. After 3 h from the start of anesthesia, kaolin ACT exceeded 500 s and the effect of argatroban seemed to be sufficient, and as a result, nafamostat administration may not be necessary. Further clinical studies are needed to determine whether the combination of nafamostat mesilate and argatroban contributes to a decrease dose of argatroban and reduced perioperative bleeding. Celite and kaolin ACTs can be used as markers to obtain close control of the required dose of argatroban in combination with nafamostat mesilate during the surgical procedure of HIT II patients.

## Data Availability

All data generated or analyzed in this study are included in this article.

## Abbreviations

ACT: Activated coagulation time
APTT: Activated partial thromboplastin time
FFP: Fresh frozen plasma
HIT II: Heparin-induced thrombocytopenia type II
PC: Platelet concentrate
RBCs: Red blood cells
TOF: Tetralogy of Fallot
